# 

**Published:** 1888-05-05

**Authors:** 


### AMERICAN JOTTINGS.

Contributions to this Column shou'd be addressed "The Editor, The Lodge, Porchester Square, London, W."

The First Congress of American Physicians and Surgeons will be held in Washington on the 18th, 19th, and 20th of September of the present year.

The *American Anthropologist*, edited by a committee of the Washington Anthropological Society, has recently appeared for the first time. It is a magazine of nearly a hundred pages, devoted to the elucidation of all kinds of questions belonging to the science of man.

At Harvard University the question of a three or a four years' curriculum for students of medicine is still being fought out. In Great Britain four years has long been the established rule at all the Universities, and at the London and Edinburgh Universities it is becoming more and more the rule among the best students to extend the period of study to five.

It is estimated that one-half of all the drugs imported into the United States is consumed in the manufacture of patent medicines. Many of us have hitherto thought the English the greatest gulls in the world. Brother Jonathan seems to "lick creation" in patent-medicine swallowing as well as in other things.

Can this be true? "The convalescents . . . are now herded in attics in a manner disgraceful to any civilised community. . . . . In these attics are congregated so many individuals that the two thousand cubic feet of space which, according to the best authorities, is essential to each, is reduced to an average of about *three hundred feet.* When the beds are prepared for the night, there is barely room enough left to enable one to walk from one end of the room to the other. Many of the patients are compelled to sleep two in a bed." These are the assertions which an American medical journal makes concerning the Philadelphia Hospital. What have the responsible managers to say in reply?

Domestic economy is receiving great attention all over the English-speaking world. The necessity for scientific instruction in many of the domestic arts has led the authorities at the Battle Creek Sanitorium, Mich., to organise a school for systematic teaching. The course of instruction will continue twenty-five weeks, and will include daily lectures, recitations, demonstrations, and practical drills in the following subjects: Cookery, laundrying, dressmaking, general housework, household hygiene, including ventilation, heating, disinfection, renovation of sick-rooms, sanitary care of the house, purification of water, detection of food adulteration, personal hygiene, and the hygienic care of children.

On the other side of the Atlantic, it is stated on what appears to be unquestionable authority, that the change in the system of nursing which was inaugurated some time ago by the Paris Municipal Council, was one which, with every respect to the pious and devoted sisterhood, was urgently required. The nursing, as such, under the old régime was far from satisfactory, as visitors to the French hospitals can testify. The work was not really done by the sisters, who merely superintended, but mainly by more or less incompetent and untrustworthy *infirmiers*, who were no more characterised by special zeal than by extraordinary discretion. In the second place, it is said that the position occupied by the sisterhood in the hospital was based on such special privileges, rights, claims, and demands that they formed a perfect *imperium in imperio*, to the detriment of efficiency, order, and obedience. They were disqualified from assisting at confinements and various other classes of cases. The American correspondent, who had himself been a medical student in Paris for several years, goes on to add: "I must confess I cannot speak with favour of them, either as nurses or as respects their attitude towards the medical staff." It is some satisfaction to know on indisputable authority that the Paris Municipality did not lack reasons for what appeared to many to be a very inopportune and high-handed proceeding.

Do the work that's nearest,
Though it's dull at whiles,
Helping, when we meet them,
Lame dogs over stiles. —*C. Kingsley.*

### A CASE FOR HELP.

The *British Medical Journal* appeals on behalf of a medical man of good position, who has been reduced to absolute destitution through no fault of his own, for help to enable him to buy a small practice, and thus make a living for himself and his wife. A good opening at a watering-place could, it is said, be secured at very small cost, and contributions towards the purchase money will be received by Dr. Farquharson, M.P., Migvie Lodge, Porchester Gardens. The following subscriptions have been received by Dr. Farquharson, M.P.: Sir William Jenner, Bart., £2 2*s.*; Sir James Paget, Bart., £6 6*s.*; Sir Joseph Lister, Bart., £20; Sir Henry Thompson, Bart., £5; Sir Spencer Wells, Bart., £1 1*s.*; Sir Andrew Clark, Bart., £8 8*s.*; Sir Prescott Hewett, Bart., £2; Dr. Ramskill, £4 4*s.*; Dr. Broadbent, £1 1*s.*: Dr. Hare, £1 1*s.*; Dr. Farquharson, M.P., £1; British Medical Benevolent Fund, £5; A Lady (per Editor *Pall Mall Gazette*), £10.

### DEATH OF A DISTINGUISHED AMERICAN WOMAN.

Dr. Clemens Lozier, of the New York Medical College and Hospital for Women, died on Thursday night of *angina pectoris*. She was the pioneer of the movement in America for the medical education of women. She practised medicine successfully for over thirty years, and had been dean of the New York College since its establishment in 1863. She was also a leader in the female suffrage movement.

### Special Notice to Newsagents, Advertisers, and the Trade.

**CHANGE OF OFFICE AND PUBLISHER.**

Notice is hereby given that the Office of The Hospital will henceforth be at 38, Old Jewry (Cheapside end), E.C., to which address all communications should be sent. Mr. A. Doig has been appointed Advertising Agent, and Mr. J. H. S. Hanning, Manager. All Cheques and Post Office Orders should be made payable to J. H. S. Hanning, 38, Old Jewry, E.C. crossed Lloyds, Barnetts, and Bosanquets Bank (Limited).

### To Secretaries and Matrons.

The Editor will be glad to have a copy of the Regulations, and also of every Report issued from the Press of Asylums, Hospitals, Convalescent and Nursing Institutions, as well as those of other Charities, so soon as they are published, and an early copy in duplicate of all appeals for funds. Notices of Annual and other Meetings, Festival Dinners, etc., will be welcomed. All such communications should be addressed to *The Editor, The Lodge, Porchester Square, London, W.* Any superintendent, secretary, matron, or other chief official who will regularly send such documents and information will be registered to receive free of cost a cover for binding The Hospital in half-yearly volumes on sending name and address to the publisher.

THE GIRLS' FRIENDLY SOCIETY.—It seems that quite a large number of nurses belong to the above society, finding the effort made by it to provide for its members in sickness, convalescence, or incurable disease, a great blessing. This shows that nurses are not so unmindful of their future, nor so adverse to joining societies, as many people suppose. Indeed, it makes evident that, in spite of adverse circumstances, nurses have attempted to be provident, and to make provision for times of need. How gladly have many nurses welcomed the National Pension Fund.

### AMUSEMENTS WITH PROFIT FOR ALL AGES.

**Rules.**—All answers must be addressed to the PRIZE EDITOR, THE HOSPITAL, 38, Old Jewry, Cheapside, London, E.C., not later than the *first* post on Thursday next. The full name and address must in all cases be given, but a *nom de plume* may be added for publication. Only one side of the paper must be written on.

### FOR MEN AND WOMEN.

**Rule.**—Competitors can enter for *all* quarterly competitions, but no competitor may take more than *two quarterly prizes* during the year.

### Acrostic Competition.

Second quarter commenced February 25th, 1888; ends May 19th, 1888.

A PRIZE of ONE GUINEA will be given to the competitor who gains the highest number of marks for best original acrostics during the ensuing quarter. All acrostics sent in for this competition will be credited according to merit, twelve marks being the highest score given for one acrostic.

A PRIZE of HALF-A-GUINEA to be given to the competitor who gains the highest score for solution of acrostics set. *One mark* only will be given to the competitors who solve all the lights of each acrostic.

Exception will be made in the case of *quotation acrostics*, when *two marks* will be given, *one* for the correct solution of all *lights*, and *one* mark for the *source* of the quotations.

This week credit will be given for

### No. 6.—Double Acrostic.

Enchanting author! whose undying pages
Reveal the life and manners of all ages;
One work I name to gild my book with light.
Invidious choice! where all alike shine bright.

1. Soldier and hero, none the claim dispute.
2. Chieftain of fame, Lord of Arran and Bute.
3. An ancient chronicle we never scorn.
4. The youth despatched the Johnstone clan to warn.
5. She gave the slipper to Lorne's lovely maid.
6. A French philosopher whom most upbraid.
7. He who for Richard did in desert wait.
8. A gayer scene, where monarchs hold their state.
9. A man who well could win an Irish smile.
10. The ancient name of a far northern isle.
11. A field of gold—so called in courtly phrase—
But oft a field of blood in knightly days.

### Answers.

No. 5. DOUBLE ACROSTIC.

H araph A

O pti C

S lende R

P etruchi O

I ncubu S

T apperti T

A lkal I

L aconi C

Correct answers have been received from Sycamore, Reldas, Lady Clara, Dunce.

### THE WORD COMPETITION.

Third Quarterly Competition commences
May 5th, 1888; ends July 28th, 1888.

Three prizes of one guinea, 15*s*. and 10*s*. 6*d*. will be given each quarter to the three competitors who obtain the highest number of marks by making the largest number of words derived from the word or phrase set for anatomical dissection; that for this, the *first* week of the quarter, being "BROAD ARROW."

OBSERVE.—Proper names, abbreviations, foreign words, words of less than four letters, and repetitions are barred; plurals, and past and present participles, of verbs are allowed. Nuttall's *Standard* dictionary only to be used.

N.B.—All words sent in must be arranged *alphabetically*, with correct total affixed.

### Results of Twelfth Week.

*The Quiver.*—Ellice (47), Dunce (41), Patience (41), Gretta (37), Sym (36), Lauriston (34), Lightowlers (34), Oliver (28), Butterfly (25).

### LITERARY COMPETITION.

This quarter the above competition of "Letters from Across the Seas," will be discontinued.

### Twelfth Week.

### Letters from Across the Sea.

*Oliver* sends the following extract of a letter from Calcutta:—

We have some friends staying with us on their way to England from the Mofussil. They have brought with them, as an attendant on one of the children, an old bearer, who is a source of great amusement to us. The first evening, when we came in from our drive, he rushed up to his mistress and said in Hindustani, "Mem-sahib, I can't stay here; I must go away. There is magic in this house. I am afraid to stay." When he was sufficiently calmed to be able to explain, he said, "When it grew dark, one of the servants came into the room; he had no lamp, no oil, not even a candle—all he had was a box of matches. He struck a match, and held it to a stick in the wall, and looked at it, and the light came, and it is burning now; and when I tried to blow it out, it wouldn't go out; and I can show it to you, mem-sahib, if you don't believe me." So we all went to see, and found that the cause of his alarm was the gas. Then we told him that was nothing, that if he went to the Eden Gardens the next evening he would see a lamp that lighted itself. He made a very low salaam, and said, "I am a very ignorant old villager, I know, but you can't make me believe all that." The next evening he went to the Gardens, and the other servants told us he mounted guard over one of the electric lamps, and drove away everyone that came near it. When the lamp lit itself, his wonder and astonishment knew no bounds. Nothing surprised him after that. When we showed him the giraffe and the kangaroo at the Zoo, he would not believe they were real, but insisted that they were moved by clockwork, and were made by order of the Government for the amusement of the people. Even when the giraffe ate bread out of his hand, he only said, "The English are a wonderful people to make a toy eat as if it were alive." He has seen a great many of the large cities in the north of India, but he despises them now after Calcutta.

EXTRACT OF A LETTER FROM NEW ORLEANS.

*Continued from page 52.*

Now for the French quarter. That is, as I told you, northward and eastward. Scores and scores of narrow streets of small houses stretch on all sides—streets so narrow that it is impossible for a street car to run in them; and even where the streets are wide enough for that conveyance, the balconies of the houses on either side stretch over the top of the car. As far as I know, this is true of every street in the French part but two—Esplanade and Elysian Fields. Now and then a house has a beautiful flowery court and numbers of rose gardens, so lovely and luxuriant that even at this early season the praise is too tame to say they are lovely. All the Creoles live here, and most of the Africans. At the corners of the streets stand groups of little octoroons and quadroons with trays of delicacies. Negro men and women stand any distance within five hundred yards of any church (of which there seem to be hundreds) offering candles, rosaries, and flowers for offerings. Beggars are numerous. The number of blessings they will give at the small cost of a nickel is marvellous. Some of them will even bless you for nothing, but these are scarce. There are several grand houses in this quarter, with terrace on terrace of flowers. In this quarter is the French market. Everyone gets up at five a.m. to go there. Everything is wonderfully cheap, and everything is to be bought. On Sunday morning crowds of people breakfast there. The dinners (to use an American phrase) are 'elegant.' Fruit, flowers, vegetables, meat, fish, clothing, pictures, rosaries, crosses, books of devotion, everything you can think of from a pin to a crocodile, is here bought and sold. A fine-looking Frenchman was doing a splendid trade in strawberries when we were there (thermometer, yesterday 75 deg., to-day 78 deg.). The noise is deafening. I believe the Frenchwomen are worse than the men or negroes. Of course French is spoken by everyone; they cry their wares and prices in French, and their only English word is evidently 'cheap,' which is useful as showing the American character. I spoke of the open drain. The reason of this terrible nuisance is that the river is higher than the city. Sometimes one can see this water moving, but not often. When it does so, it flows into a wide open ditch, or bayou, called St. John's, and into some cypress swamps round the lake. This is the theory, but it hardly answers in practice. If it does not run there, it ought to, which consolation seems to be quite enough for the American—*i.e.*, southern—mind. Next time I hope to describe the churches.

### THE CHILDREN'S CORNER.

### Puzzles—First Week.

No. 1. RIDDLE-ME-REE.

My *first* is in ocean, but not in waves;
My *second* is in river, but not in caves;
My *third* is in boatman, but not in row;
My *fourth* is in ebb, but not in flow.

No. 2. DECAPITATIONS.—I am a quick pain; behead me, and I am a market; behead me again, and I am a profession.

No. 3. GEOGRAPHICAL CHARADE.—My first soon stops short in "constancy"; my second frequently stands for nothing; but my whole is a province in Ireland.

No. 4. DOUBLE ACROSTIC.—Initials give the Christian name, the finals the surname of a famous novelist. 1. A town in Derbyshire famous for various manufactures. 2. The largest of the Sandwich Islands. 3. An European sea. 4. A town in Holland where an important treaty was signed. 5. A Swiss lake. 6. The birth-place of a famous German Reformer. 7. A town in France famous for its china.

### Results of Third Week.

No. 9. DIAMOND PUZZLE.

M
G A D
A R C O T
D N I E P E R
M A C E D O N I A
A N D O V E R
B E N I N
A I X
A

No. 10. CHARADE.—Jack-an-apes = Jackanapes.

No. 11. GEOMETRICAL PUZZLE.

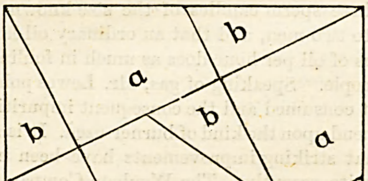

No. 12. DOUBLE ACROSTIC.

L as H
O hi O
R o W
D ac E

*All right.*—A. C. K., McCulloch, Bunny, Nunkey. *Three right.*—Judy, Happy Eliza, Spider, Dame Durden, Hercules. *Two right.*—Scrooge, Mount Edgecumbe, Gem, Good Old Killaloe. *One right.*—Plummy Fluff.

